# Stable and high expression of Galectin-8 tightly controls metastatic progression of prostate cancer

**DOI:** 10.18632/oncotarget.17963

**Published:** 2017-05-18

**Authors:** Lucas Daniel Gentilini, Felipe Martín Jaworski, Carolina Tiraboschi, Ignacio González Pérez, Monica Lidia Kotler, Anne Chauchereau, Diego Jose Laderach, Daniel Compagno

**Affiliations:** ^1^ Molecular and Functional Glyco-Oncology Laboratory, IQUIBICEN-CONICET, Departamento de Química Biológica, Facultad de Ciencias Exactas y Naturales, Universidad de Buenos Aires, Ciudad de Buenos Aires, Argentina; ^2^ Laboratorio de Disfunción Celular en Enfermedades Neurodegenerativas y Nanomedicina, IQUIBICEN-CONICET, Departamento de Química Biológica, Facultad de Ciencias Exactas y Naturales, Universidad de Buenos Aires, Ciudad de Buenos Aires, Argentina; ^3^ Institut Gustave Roussy-INSERM U981, Villejuif, France; ^4^ Departamento de Ciencias Básicas, Universidad Nacional de Luján, Luján, Argentina

**Keywords:** prostate cancer, galectins, anoikis resistance, metastasis, tumour microenvironment

## Abstract

Two decades ago, Galectin-8 was described as a prostate carcinoma biomarker since it is only expressed in the neoplastic prostate, but not in the healthy tissue. To date, no biological function has been attributed to Galectin-8 that could explain this differential expression. In this study we silenced Galectin-8 in two human prostate cancer cell lines, PC3 and IGR-CaP1, and designed a pre-clinical experimental model that allows monitoring the pathology from its early steps to the long-term metastatic stages. We show for the first time that the natural and conserved expression of Gal-8 in tumour cells is responsible for the metastatic evolution of prostate cancer. In fact, Gal-8 controls the rearrangement of the cytoskeleton and E-Cadherin expression, with a major impact on anoikis and homotypic aggregation of tumour cells, both being essential processes for the survival of circulating tumour cells during metastasis. While localized prostate cancer can be cured, metastatic and advanced disease remains a significant therapeutic challenge, urging for the identification of prognostic markers of the metastatic process. Collectively, our results highlight Galectin-8 as a potential target for anti-metastatic therapy against prostate cancer.

## INTRODUCTION

Prostate cancer (PCa) is a major problem of health, and it is currently the second most prevalent cancer in men after lung cancer. In fact, PCa incidence and mortality represent respectively 30.6 and 7.8 out of 100,000 total cancer cases (IARC, WHO) [1]. However, its incidence continues to rise due to higher life expectancy and subsequent population ageing. The association of surgery, radiation treatments and androgen ablation are effective against localized prostate tumours [2]. However, between 15 and 20% of patients with PCa evolve into advanced stages of the disease developing bone and lymph node metastases. There are no effective treatments for these stages, mainly because tumour growth becomes resistant to castration or taxane-based treatments. Therefore, in-depth understanding of the molecular mechanisms involved in the transition from early towards advanced phases of PCa will allow an earlier diagnosis and consequent prevention of metastasis.

Effective cancer therapies typically capitalize on molecular differences between healthy and neoplastic tissues that can be targeted with drugs [3]. Glycans abundantly decorate the surface of all mammalian cells, and the extracellular matrix with which they interact [4]. It has been recognized that the structure of cell surface glycans can change under different physiological and pathological conditions. Particularly, malignant transformation is associated with the synthesis of altered glycan determinants in the tumour microenvironment [5].

The responsibility of decoding the information displayed by changes in glycan structures is attributed to endogenous glycan-binding proteins or lectins. In particular, galectins are a family of evolutionarily conserved glycan-binding proteins characterized by their affinity for N-acetyllactosamine sequences which can be displayed on cell surface glycoconjugates [6–7]. Galectins are involved in the regulation of different cellular processes and their expression is altered during several pathological conditions including cancer and autoimmunity [6, 8]. A series of studies in experimental models and cancer patients have reported significant associations among the expression of galectins and tumourigenesis, metastatic potential and tumour-immune escape [9–10]. In most cases, expression of galectins in the tumour microenvironment is associated with a poor clinical outcome [6].

We have recently showed a regulated pattern of expression of galectins along PCa progression, showing that Gal-1 is the most expressed member of this family and the only one that is progressively increased throughout the evolution of the disease, Gal-3 is decreased and turned off at advanced stages of the disease, and Gal-8 is highly expressed but does not show any regulation during PCa progression [11], making it a suitable candidate for targeting throughout the entire disease.

Interestingly, Gal-8 is ubiquitously expressed in normal tissues except the prostate. Indeed, Gal-8 was firstly named PCTA-1 (Prostate Cancer Tumour Antigen-1) since it was initially identified as being highly expressed in neoplastic areas, and absent in the healthy compartment of the same samples of PCa patients [12]. This galectin has both pro- and anti-adhesive functions depending on its subcelular localization [8, 13–14]. However, intracellular Gal-8 shows anti-proliferative and pro-apoptotic functions by promoting cell-cycle arrest [13, 15]. Gal-8 also controles the immune response and plays a crucial role in inflammation and autoimmunity (thoroughly reviewed in [16]). In megakaryocytes, Gal-8 allows the internalization of the coagulation factor V, which is critical not only for the coagulation cascade but also for the maturation of platelets [17]. It is currently well known that platelets foster the metastatic process by protecting circulating tumour cells and tumour cell transendothelial migration [18]. Taken altogether, these findings suggest Gal-8 plays a key role in PCa cell dissemination. Although there is partial evidence in literature, the role of Gal-8 in prostatic carcinogenesis remains inconclusive and required further research.

In this regard, we developed a novel animal model based on the stem-like IGR-CaP1 cell line to evaluate all the stages of the human disease. Although neither PCa tumourigenesis nor primary tumour growth is controled by Gal-8 expression, we demonstrate for the first time the key role played by Gal-8 in the metastatic process controlling the actin filament reorganization and filopodia formation and E-Cadherin expression, which in turn allow effective migration and survival of circulating tumour cells along the metastatic process.

## RESULTS

### Gal-8 is expressed by all human PCa cells lines

**Figure 1:**
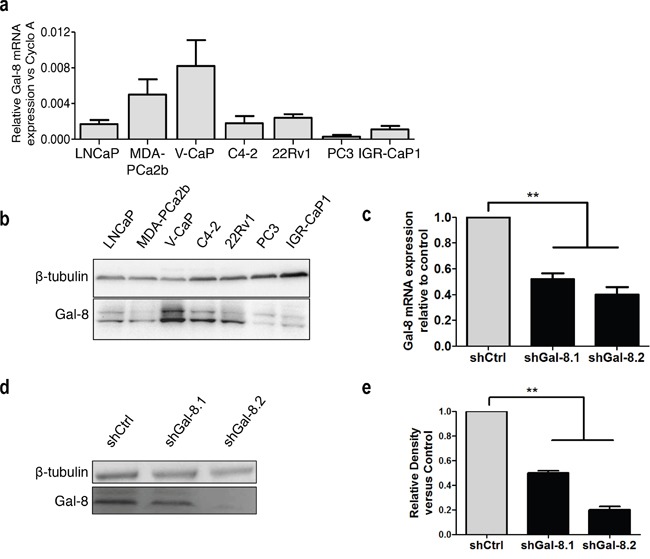
Galectin-8 expression profile in human PCa cell lines and metastatic model of prostate cancer **(a)** Expression of galectin-8 mRNA by real-time RT-PCR in several PCa cell lines. Results are expressed as galectin mRNA relative to Cyclophilin A as housekeeping gene. **(b)** Immunoblotting analysis of galectin-8 expression in PCa cell lines. **(c-d)** Real time RT-PCR (c-) and western-blotting **(d-e)** of Gal-8 expression in IGR-CaP1 cells transduced with a control-shRNA or two different Gal-8-shRNA -expressing LV (shGal-8.1 or shGal-8.2). **(e)** Mean of relative band density obtained in at 4 independent immunoblots. ** p<0.005.

We first verified the expression of Gal-8 in several human PCa cell lines by real time RT-PCR (Figure 1a) or western blot (Figure 1b). These results demonstrate that all human PCa cells tested express this galectin, as our group and others previously showed [11–12, 19]. Since we have already demonstrated high oncogenic and metastatic properties of IGR-CaP1 cell line (IGR-CaP1) [20–21], and since PC3 cell line (PC3) is one of the most used models for PCa studies, we decided to use both in our further exploration of the role of Gal-8 in the PCa progression.

### Gal-8 controls PCa cell migration and aggregation but not their proliferation or adhesion to endothelial cells

**Figure 2:**
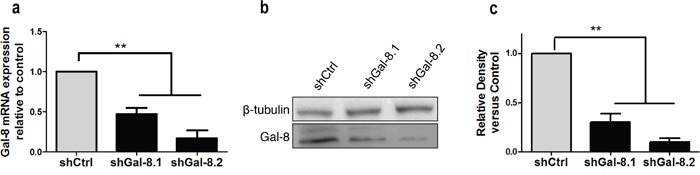
Silencing of Gal-8 in PC3 **(a)** Real time RT-PCR, **(b)** western blotting to analyze the Gal-8 mRNA **(a)** and protein **(b)** expression in PC3 cells transduced with a control-shRNA (shCtrl) or two different Gal-8-shRNA -expressing LV (shGal-8.1 or shGal-8.2). **(c)** Mean of relative band density obtained in at 4 independent immunoblots. ** p<0.005.

Metastasis is a multifactorial process influenced by i) tumour cell migration properties; ii) homotypic cell aggregation that provides support to withstand blood vessel circulation-associated stress; and iii) adhesion of tumour cells with the endothelium. To evaluate whether Gal-8 is controlling these oncogenic parameters of human PCa cell lines, we transduced IGR-CaP1 and PC3 independently with two different lentivirus (LV) expressing GFP and a human Gal-8 shRNA (shGal-8.1 and shGal-8.2) or a control shRNA (shCtrl) containing a sequence that does not show nucleotide complementarity with any sequence found by BLAST in the human genome. After purification of transduced GFP^+^-IGR-CaP1 or -PC3 cells by cell sorting, we analyzed the level of specific knock-down of Gal-8 expression. Gal-8 mRNA expression in transduced IGR-CaP1 was reduced by 50% and 60% with shGal-8.1 and shGal-8.2 respectively (Figure 1c). In the same way, Gal-8 protein level was reduced by 50% to 80% with the shGal-8.1 and shGal-8.2 respectively (Figure 1d–1e) with high specificity since Gal-1 (Supplementary Figure 1), another member of the galectin family, was not significantly knocked-down by both Gal-8 shRNA used here. Higher efficiencies were obtained with both shGal-8 used to silence the expression of Gal-8 in PC3 (Figure 2).

**Figure 3:**
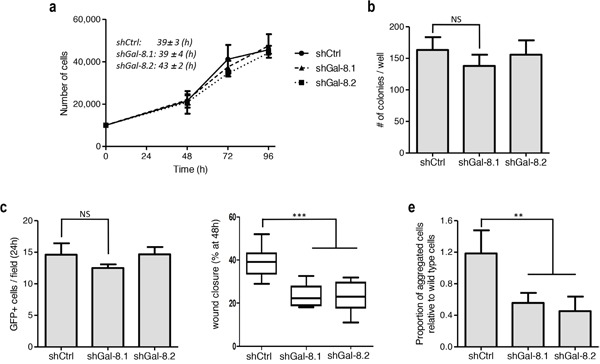
Silencing of Gal-8 impacts migration and homotypic aggregation while does control neither proliferation and clone formation nor adherence to endothelial cells **(a)** Effect of the silencing of Gal-8 on the proliferation of IGR-CaP1 cells. Doubling times were calculated for each cell line on three independent experiments and expressed as mean ± SD. **(b)** Colony formation assay was performed and the number of colonies was counted. **(c)** PCa cells (GFP+ cells) ability to adhere to endothelial cells. The number of GFP+ tumour cells was counted per field. **(d)** Migration of IGR-CaP1 cells were analyzed by a wound healing assay, data are presented at 48h after scratch. **(e)** Homotypic aggregation of tumour cells as indicated in Material & Methods. Data are presented as mean ± SD of at least 3 independent experiments. NS: no significative difference; ** p<0.005; *** p< 0.0005.

**Figure 4:**
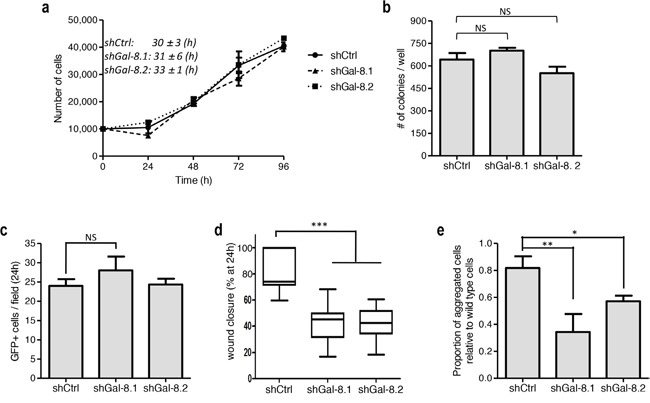
Silencing of Gal-8 impacts migration and homotypic aggregation while does control neither proliferation and clone formation nor adherence to endothelial cells of PC3 cells **(a)** Effect of the silencing of Gal-8 on the proliferation of PC3 cells. Doubling times were calculated for each cell line on three independent experiments and expressed as mean ± SD. **(b)** Colony formation assay was performed and the number of colonies was counted. **(c)** PCa cells (GFP+ cells) ability to adhere to endothelial cells. The number of GFP+ tumour cells was counted per field. **(d)** Migration of PC3 cells were analyzed by a wound healing assay, data are presented at 24h after scratch. **(e)** Homotypic aggregation of tumour cells as indicated in Material & Methods. Data are presented as mean ± SD of at least 3 independent experiments. NS: no significative difference; * p<0.05; ** p<0.005; *** p< 0.0005.

We thus wondered whether flaws in any of oncogenic properties could be observed when Gal-8 is silenced. For this reason, we first analyzed cell proliferation and found no significant differences in cell doubling time (39-43h for the three conditions) (Figure 3a). In the same way, no differences were found when we analyzed the colony forming capacity (Figure 3b) or the tumour cell adhesion to endothelial cells (Figure 3c). However, silencing of Gal-8 significantly decreased wound closure (covered wound area after 48h: 23±5%, 26±8% versus 38±8%, shGal-8.1, shGal-8.2 and shCtrl respectively; (Figure 3d, and Supplementary Figure 2). We then analyzed whether silencing of this galectin could decrease homotypic aggregation, a mechanism that promotes survival of tumour cells while circulating in the blood. Indeed, we found that homotypic aggregation of IGR-CaP1 cells was also impaired when Gal-8 was silenced (Figure 3e, and Supplementary Figure 3), suggesting Gal-8 as a protein that controls positively the metastatic process of PCa cells. Same results were obtained silencing Gal-8 in PC3 (Figure 4). Altogether, our results strongly suggest that Gal-8 finely tunes both migration and homotypic aggregation, both actively involved in the metastatic progression of PCa.

### Prostate cancer metastatic model based on subcutaneous injection of IGR-CaP1

**Figure 5:**
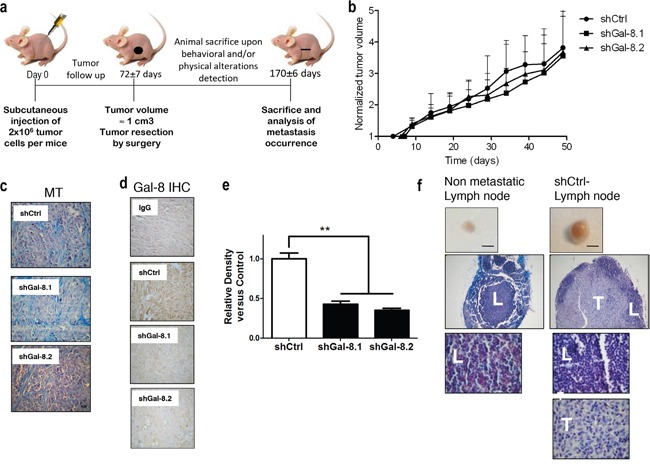
Silencing of Gal-8 effect on tumourigenic and metastatic ability of IGR-CaP1 cell line **(a)** Scheme of the experimental metastatic model developed with IGR-CaP1 cell line in athymic male mice. **(b)** Analysis of the IGR-CaP1 tumour growth. The values are expressed normalized to the first day of the tumour exponential growth. **(c)** Histological analysis reveals that tumour morphology was unaltered when Gal-8 was silenced compared with the control cells. **(d)** GaI-8 is still efficiently silenced in the primary IGR-CaP1 tumour. Immunohistochemistry against galectin-8 in control (shCtrl) and Gal-8 (shGal-8.1 or shGal-8.2) silencing tumours. Corresponding isotype antibody (IgG) is used as negative control. **(e)** Relative density analysis was performed as the mean ± SD of 5, 7 and 8 samples, for shCtrl, shGal-8.1 and shGal-8.2 condition respectively. ** p<0.01 (magnification, x40). **(f)** Analysis of long-term spontaneous metastasis to draining lymph nodes obtained from mice after resection of primary subcutaneous tumour. Representative images of normal or metastatic lymph nodes are shown. Scale bar: 2.5 mm. Histological analyses by modified Masson Trichrome staining were performed to confirm the presence of carcinoma invading tissue (magnification, x10 and x100). L: lymphocytes, T: prostate tumour cells.

Transition from benign to malignant tumours is a multi-event process that includes loss of checking point controls of the cell cycle, activation of oncogenes or inactivation of tumour suppressor genes, loss of sensitivity to programmed cell death, such as apoptosis or anoikis, altered morphology and fewer contacts with neighbouring cells and the extracellular matrix components, and an ultimate capacity to disseminate to different tissues and form secondary tumours (i.e. metastatic disease). In the case of prostate, several preclinical models use xenotransplants (injection of human cells into immunodeficient mice) and, in this case, an important limitation refers to the fact that few human PCa cell lines exist and most of them were isolated from metastasis samples. Under such scenario, the analysis of prostate tumourigenesis is biased because it lacks the initial steps [22]. Thus, we decided to use IGR-CaP1 for the generation of a new metastatic animal model because this cell line had not already undergone the metastatic selection in the original patient as the PC3 cell line did. Figure 5a illustrates the protocol we optimized to study the development of PCa primary tumour to metastatic stages and detailed in Materials and Methods. As results, the IGR-CaP1-shCtrl based preclinical model resulted in 83% of injected mice developing tumours within one week. Tumours exhibited a growth doubling time of 23±9 days, and all the mice showed inguinal lymph node metastasis 170±6 days after tumour cell injection (Table 1). Of note, only one mouse showed motor impairment, disabling this mouse to feed itself, hence we decided to sacrifice all the mice at this point. Only the draining lymph node metastasis was observed, and neither lung nor bone metastatic nodes were detected at this time. To verify that no metastatic lymph nodes do not present any presence of tumour cells, we analyse the presence or not of the GFP gene performing PCR analysis of DNA extract. As results, only draining lymph node showed metastasis at this time of sacrifice and no GFP gene identification was found neither in shGal-8.1 nor in shGal-8.2 tumour injected mice (data not shown).

**Table 1:** Effect of Gal-8 knock-down on physio-pathological parameters of IGR-CaP1 prostate cancer as a metastatic experimental model

Cell lines	Tumourigenecity	Latency (days)	Duplication time (days)	Metastasis frequency (%)
IGR-CaP1 shCtrl	83% (5/6)	4 ± 6	23 ± 9	100% (4/4)
IGR-CaP1 shGal-8.1	88% (7/8)	7 ± 3	20 ± 5	0% (0/7)
IGR-CaP1 shGal-8.2	80% (8/10)	6 ± 3	22 ± 6	0% (0/8)

### Galectin-8 does not influence prostate cancer primary tumourigenesis but tightly controls metastasis development

Two decades ago, Gal-8 was described as a prostate tumour marker since it is highly expressed in neoplastic tissue and barely expressed in the normal prostate [11–12]. We thus subcutaneously injected athymic nude mice with transduced IGR-CaP1 cells to assess whether pathological parameters were altered by specifically silencing Gal-8. The results in Table 1 show the same frequency of mice developing tumour (88% with the IGR-CaP1 shGal-8.1, 80% with the IGR-CaP1 shGal-8.2 versus 83% with the shCtrl), and no significant differences were found neither in the time span required to detect palpable tumours (i.e. the latency period) nor in tumour growth (Table 1: duplication time, Figure 5b). Histological analysis revealed that primary tumour morphology was unaltered when Gal-8 was silenced compared with the control cells (Figure 5c), and the expression of Gal-8 in primary tumours was still efficiently down-regulated in shGal-8.1 or shGal-8.2 conditions compared to the shCtrl, as determined by immunohistochemistry after tumour resection (Figure 5d–5e). We then analyzed the draining lymph nodes of injected mice, and showed a normal lymphatic morphology in shGal-8.1- and shGal-8.2-mice and a highly undifferentiated and non metastatic phenotype in shCtrl-mice (Figure 5f). Altogether, our results strongly suggest that Gal-8 does not control the tumourigenicity of human IGR-CaP1 cell line, given the same frequency of tumour-bearing mice was observed in both experimental groups. However and more importantly, when we analyzed lymph node metastasis frequency, silencing of Gal-8 totally abolished the metastatic potential of IGR-CaP1, showing that this galectin would control the progression of PCa to advanced-metastatic stages of the disease.

### Gal-8 acts on cytoskeleton integrity and anoikis resistance through maintaining E-Cadherin expression

**Figure 6:**
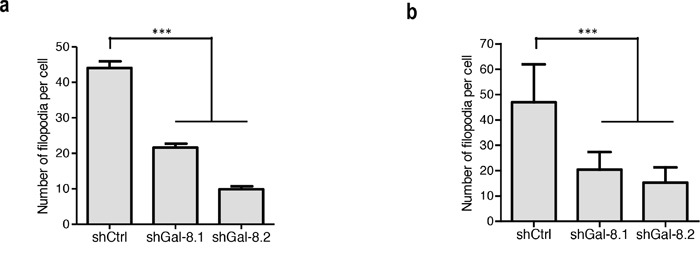
Gal-8 knock-down decreases the cytoskeleton reorganization Analysis of filopodia formation by staining the actin cytoskeleton with rhodamin-conjugated phalloidin on PCa cell lines (representative photographs are shown in Supplementary Figure 4). Quantification of the number of filopodia per cells was performed based on (at least 100 cells were analyzed per condition). **(a)** in IGR-CaP1 and **(b)** in PC3. *** p< 0.0005.

**Figure 7:**
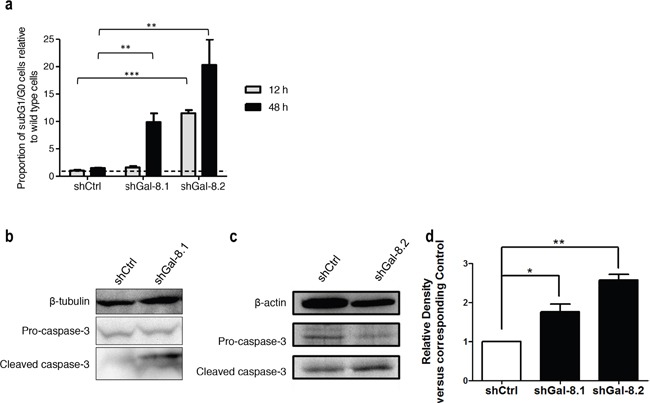
Gal-8 knock-down decreases the anoikis resistance and promotes apoptosis in suspension culture condition **(a)** After cultured in poly-HEMA coated dishes for indicated times, the percentage of sub-G1/G0 cells was analyzed and proportion of subG1 cells are indicated for each cell line, comparing two different time after plating (12 and 48h). **(b-c)** Immunoblotting for apoptosis markers after 12 h of culture in poly-HEMA coated dishes were performed. Quantification of the band intensity relative to shCtrl is indicated. **(d)** Data of quantification are represented as the mean of at least 3 independent experiments, * p<0.05 ** p<0.01; *** p<0.005.

**Figure 8:**
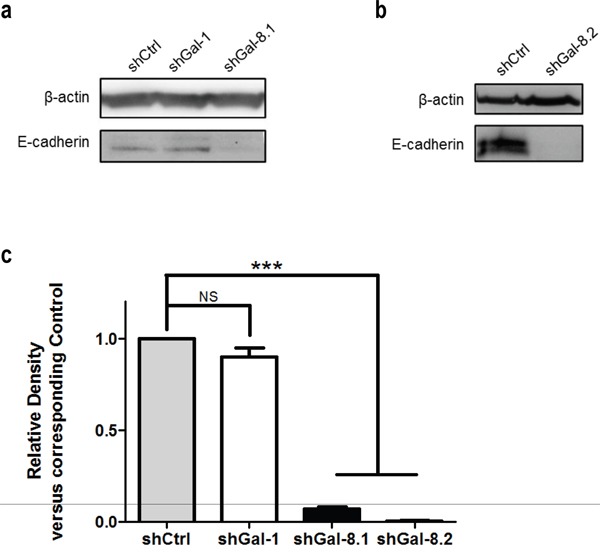
E-Cadherin expression is correlated with Gal-8 expression in IGR-CaP1 **(a-b)** Expression of E-Cadherin in control and Gal-8 knock-down IGR-CaP1 cell lines was analysed by immunoblotting in the protein total extract of all cell lines. A specific Gal-1-shRNA-expressing LV (shGal-1) (Laderach DJ et al. Cancer Res. 2013) was used to verify the specificity of the E-Cadherin decrease by Gal-8 knock-down. Quantification of the band intensity relative to shCtrl is indicated. a- IGR-CaP1 shGal-8.1 and control with a shGal-1 to show specificity of the effect, b- IGR-CaP1 shGal-8.2. **(c)** Relative density analysis of 3 independent immunoblots.

**Figure 9:**
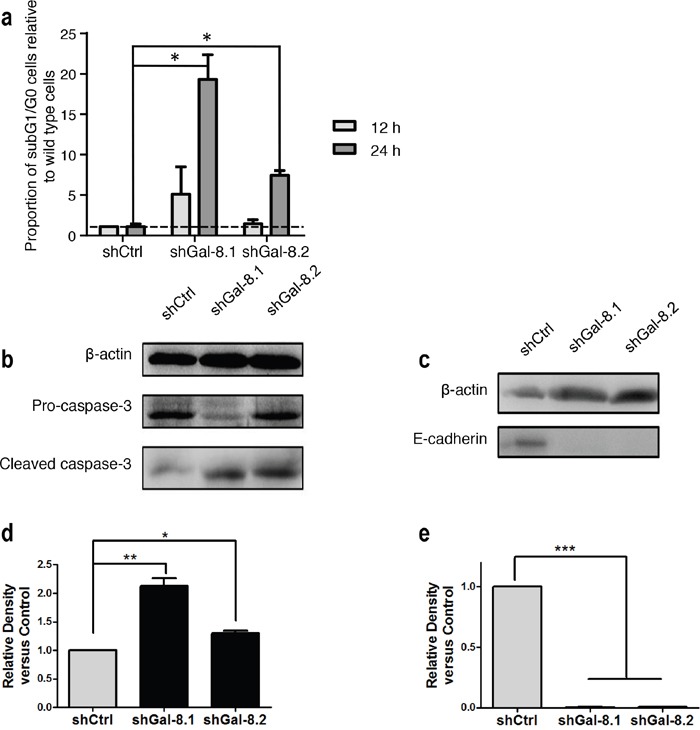
Gal-8 knock-down decreases the anoikis resistance and E-Cadherin expression in PC3 cells **(a)** After cultured in poly-HEMA coated dishes for indicated times, the percentage of sub-G1/G0 cells was analyzed. Proportion of subG1/G0 cells are indicated for each cell line. **(b)** Immunoblotting for pro-caspase-3 and cleaved caspase-3 as apoptosis markers after 12 h of culture in poly-HEMA coated dishes were performed. **(c)** Expression of E-Cadherin in control and Gal-8 knock-down PC3 cell lines. **(d)** Quantifications of the band intensity corresponding to immunoblot c- relative to shCtrl are indicated, and **(e)** the quantification of the band intensity relative to shCtrl are indicated by performing 3 independent immunoblotting of protein total extracts from all cell lines. Data are presented as mean ± SD of at least 3 independent experiments. * p<0.05; ** p<0.01; * p< 0.0005.

Tumour cell migration is a key step in tumour dissemination; changes in the actin cytoskeleton structure play a major role in the control of cell motility. To determine whether Gal-8 could decrease cytoskeleton reorganization and thus controls PCa metastasis, cells were stained with Rhodamine-conjugated phalloidin to analyze filopodia formation. As shown in Figure 6a–6b, silencing of Gal-8 sharply reduced filopodia development of IGR-CaP1 cells (22±1, 10±1 versus 44±2 filopodia per cell in shGal-8.1, shGal-8.2 and shCtrl-cells respectively), which may explain the defect on cell migration previously observed. As homotypic aggregation promotes the survival of tumour cells in the blood stream, we then analyzed the resistance to anchorage-dependent apoptosis (i.e. anoikis) since it is an essential property to face blood pressure. We therefore cultured tumour cells in suspension, preventing cell adhesion by using polyHEMA-treated cell culture dishes. Under these conditions, shGal-8-cells clearly showed an induction of apoptosis (Figure 7a), since the percentage of cells in subG1/G0 phases of the cell cycle are increased with a time-dependency only when Gal-8 was silenced when compared to shCtrl-cell lines (Figure 7a). To verify whether Gal-8 impacts upon the resistance to anoikis, we studied caspase-3 cleavage in all cell lines, and found that Gal-8 silencing promoted the cleavage of this caspase, further supporting the anti-apoptotic role of this lectin in PCa tumour cells (Figure 7b–7d). Finally, E-Cadherin is a transmembrane glycoprotein that participates in cell-cell adhesion controlling the integrity of epithelial cells [23]. We studied its expression levels in both cell lines and found that Gal-8 knock-down dramatically down-regulated the expression of E-Cadherin, since its protein levels were reduced more than 10-fold or was undetectable in IGR-CaP1 shGal-8.1 (Figure 8a) or shGal-8.2 (Figure 8b) respectively compared to control, while silencing of Gal-1 did not significantly alter its expression levels (Figure 8a–8c). All the same results were obtained when we used PC3 (Figure 9), Thus, we postulate the control of E-Cadherin expression as a critical mechanism through which Gal-8 can foster homotypic aggregation and in turn allow anchorage-independent cell survival of disseminating tumour cells.

## DISCUSSION

In this study, we addressed the role of Gal-8 produced by the tumour in the tumourigenesis and metastasis of PCa using two different human cell lines. Tumour development is associated with extensive modifications of the glycosylation of cell surface glycoconjugates that are docking sites for endogenous galectins. Particularly in PCa, it was reported in 1996 that Gal-8 is exclusively expressed at the neoplastic prostate tissue, and PCTA-1 – as Gal-8 was then known- is postulated as a new prostate cancer marker [12]. However, Gal-8 was so far one of the least appreciated galectins, and to date no functional study has focused on the role of this galectin neither in prostate carcinogenesis nor in the evolution of this pathology. Herein, we showed that silencing of Gal-8 in tumour cells does not interfere with proliferation and colony formation in both human PCa cell lines, strongly suggesting that this galectin does not act on cell growth. Moreover, human PCa cells are still capable to generate tumours when Gal-8 knocked-down cells are subcutaneously injected into athymic nude mice. Specific Gal-8 silencing in the tumour cells did not alter tumour latency, tumour take or tumour growth. These results indicate that the sole expression of Gal-8 by tumour cells at neoplastic stages cannot account for prostate cell tumourigenecity. Nonetheless, we cannot exclude this galectin participates in the malignant transformation of normal prostate cells. Undoubtedly, it would be interesting to both assess whether these cells express Gal-8, and evaluate if ectopic expression of this lectin increases tumourigenesis *in vivo*. Irrespective of its role in the tumourigenic process we show here for the first time that Gal-8 tightly controls the migratory and metastatic properties of prostate tumour cells.

Tumour metastasis is a multi-step process involving several cellular and molecular interactions. Recognition of glycoconjugates by galectins regulates tumour behaviour through both intrinsic and extrinsic signals [9]. Galectins regulate also cell-cell and cell-extracellular matrix (ECM) interactions, with different members of the family eliciting particular and sometimes antagonist effects. In fact, Gal-1 increases, while Gal-8 reduces tumour cell-ECM interactions [13, 24]. Altogether, these effects may contribute to a favourable tumour microenvironment and allow distant dissemination of transformed cells. Despite these observations, there are relatively few *in vivo* studies addressing the origin and function of galectins and exploring these phenotypes in prostate cancer [25–26]. In fact, expression levels of galectins-1 and -3 were reported to be associated with the growth and metastatic properties of prostate tumours, and may correlate with a poor prognosis [25, 27–28]. Galectin-3 is the first member of the family, which function has been addressed using a rat experimental models [27, 29–31]. However, these results reveal indirectly a potential role played by Gal-3 in the formation of metastases in this unique animal model, but not in patients with advanced disease when this galectin is not longer expressed. Recently, Gal-4 upregulation was also described as pro-metastatic factor for metastasis in PCa [32]. As the results, tumours growth faster in mouse after this process of experimental selection or exogenic upregulation system. Thus, the increase of Gal-4 is more likely to have a strong influence on the proliferative properties of the artificially selected cells rather than on the metastatic potential of PCa cell lines [32]. Since such increase of Gal-4 at the protein level does not occur naturally neither during the disease progression nor in the majority of high grade patients, and since Gal-3 expression is shutting down in the more aggressive PCa tumours [11, 32], our results strongly suggest that Gal-8 is likely the unique galectin that controls the metastatic process in patients.

To study the role of Gal-8 in prostate tumourigenesis, there was needed for a PCa model that faithfully recapitulates the phenotypic and molecular events occurring along the human disease. To date, such a model did not exist [22]. We thus decided to design an experimental model to monitor the pathology from its early steps to long-term spontaneous metastases. For this proposal, we chose the IGR-CaP1 that expresses Gal-8 as well as a large number of cancer stem-cell markers [21], which suggested a high potential of tumour spreading as shown by earlier published data. In the IGR-CaP1 preclinical model we previously used [20–21], neither visceral nor bone metastasis were obtained using orthotopic injections; and only intra-cardiac or intra-bone injection allowed bone metastasis. However, these inoculation routes do not recapitulate all the steps of the metastatic process, as cells undergo a wide range of molecular changes at the primary site that in turn has a major impact upon migration and invasion through the extracellular matrix and the endothelial compartment. We thus decided to test whether the surgical resection of subcutaneous IGR-CaP1 tumours led to long-term metastasis establishment. Using this protocol we observed metastasis in draining lymph nodes in all the mice that had been injected and surgically intervened. We provided then evidence that silencing of Gal-8 in human PCa cell lines abolished tumour migration to draining lymph nodes, as the first step for tumour dissemination. Although not involved in the tumourigenesis of prostate tumour cells, our study identifies for the first time the role of Gal-8 in metastasis establishment in PCa.

Several studies have already shown that Gal-8 function depends upon its sub-cellular localization [13, 15, 33]. In PCa, we show here for the first time that the expression of Gal-8 by tumour cells is essential to generate the required conditions for effective metastasis. As already shown for other galectins, namely Gals-1 and -3 in other human carcinomas [34–37], the present study suggests that Gal-8 may also induce cytoskeleton reorganization, with a profound impact on the promotion of homotypic aggregation and consequent protection against apoptosis induced by the loss of cell anchorage and E-Cadherin expression. All these parameters determine tumour cell ability for effective migration and metastasis development. Interestingly, we recently showed that Gal-1 has pro-angiogenic effects in PCa [11], and is associated with the development of metastasis in human hepatocellular carcinomas by triggering EMT and down-regulation of E-Cadherin [38]. Herein, we show that, in contrast with Gal-8, silencing of Gal-1 does not impact upon E-Cadherin expression, suggesting tissue-specific pro-metastatic effects of each members of the galectin family.

Even though the reasons why Gal-8 is solely expressed in the neoplastic prostate and its implications in prostate carcinogenesis still leave some interesting unanswered questions, we show here for the first time that controlling the expression of Gal-8 was enough to completely abolish metastasis development of a human PCa cell line with highly-expressed cancer stem cell markers. Our data could provide further insight into the design of new therapeutic strategies, by targeting this galectin to prevent the dissemination of PCa cells, and render new curative perspectives for advanced-PCa patients.

## MATERIALS AND METHODS

### Cells and animals

Human PCa cell lines used included: the hormone-responsive LNCaP and MDA-PCa2B cell lines and the castration-resistant cell lines, V-CaP, C4-2, 22Rv1, PC3 and IGR-CaP1. All cells were authenticated by our laboratory by i- standard PCR and RT-PCR analyses; ii- Cell morphology evaluated by microscopic examination on a daily basis. iii-Growth properties of LNCaP and MDA-PCa2B cells were regularly tested as well through their responsiveness to androgens using MTT assay. Cells were incubated for 24 hours in phenol red-free RPMI, 10% charcoal-treated and medium was supplemented with 10% of fresh heat-inactivated FBS (PAA, Cell Culture, Austria) for 3 days before analyzing growth and gene expression; iv- Prostate-specific antigen (PSA) induction was evaluated by real-time reverse transcriptase quantitative PCR (RT-qPCR); and finally v- Routine tests for other PCa cells included examination of androgen-insensitive growth (MTT method) and PSA induction (real-time RT-PCR). The PC3 cell line was provided by E. Vazquez. Bovine aortic endothelial cells (BAEC) were provided by M.T. Elola. These cell lines were originally obtained from the American Type Culture Collection except IGR-CaP1 cells which were obtained directly from its owner Dr. Chauchereau. LNCaP, C4-2, 22Rv1, PC-3 and IGR-CaP1 cells were cultured in RPMI 1640 (Gibco). BAEC and V-CaP cells were cultured in Dulbecco's modified Eagle's medium (Gibco). Medium was supplemented with 10% (PCa cell lines) or 20% (BAEC) heat-inactivated FBS (PAA), 2 mmol/L L-glutamine, 100 mg/mL streptomycin, and 100 U/mL penicillin. BAEC were used at passage 14 or less. For the *in vivo* assays, 6-week-old nude mice were obtained from The National University of La Plata (La Plata, Argentina) and maintained in accordance with the Institutional Animal Care and Use Committee guidelines (FCEyN, Buenos Aires, Argentina).

### Lentivirus vector production and transduction of cells

pLv-HTM plasmid (provided by Trono Didier, Geneva University, Geneva, Switzerland) is a self-inactivation third generation HIV-1–derived. ShRNA cloning and lentivirus production conditions were already described in [11] (shRNA sequences, Table 2). Spin infection was then performed. Briefly, IGR-CaP1 wild type cells were trypsinized and 2×10^5^ cells were counted, placed in 3 mL polystyrene tubes, centrifuged and suspended in 0.5 ml of viral supernatants in the presence of polybrene (19.2 μg/ml). Then, this cell and virus suspension was centrifuged during 1 h at 1,200 rpm at 25°C and plated in 6-well dishes with fresh medium. After 1 week, transduced cells (GFP+) were purified using a FACSAria II cell sorter (BD Bioscience). Purification of the transduced cells was carried out if GFP^+^ cells did not exceed 20%, in order to minimize the number of viral integrations and thus guarantee a minimum perturbation of the genome.

**Table 2:** List of shRNAs targeting the different mRNA sequences

LV-shRNA	shRNA target sequence (5´→ 3´)
shCtrl	GATAGCAATGACGAATGCG
shGal-1	AGACAGCAACAACCTGTGC
shGal-8.1	GGACGAACTGTCGTCGTTA
shGal-8.2	CTAAGCAGTATTGATACACTA

### Real time RT-PCR

Transcriptional profile of galectins was analyzed in human PCa cell lines (log phase of growth) that are representative of different stages of tumor progression. RNA purification, reverse transcription reaction, qPCR conditions and data analysis were performed as previously described [11]. Cyclophilin A (Cyclo A) was used as an internal reference gene [39]. Primers sequences are listed in Table 3. Equivalent amounts of RNA were tested to rule out residual genomic DNA contamination.

**Table 3:** Primers used to determine Gal-8 expression in PCa cell lines

Gene	Primer sequence (5´→ 3´)
Cyclophilin A _ Fw	CCCATTTGCTCGCAGTATCCTAGA
Cyclophilin A _ Rev	GGCATGGGAGGGAACAAGGAAAAC
Galectin-8_Fw	CTCTGCTCTATGGCCACAGGATC
Galectin-8_Rev	GTTCGTCCAGGGCCCATGG
PSA _ Fw	AGACACAGGCCAGGTATTTCAGGTC
PSA _ Rev	CACGATGGTGTCCTTGATCCACTTC

### Immunoblotting and immunohistochemistry

Specificity of anti-galectin antibodies was previously evaluated by immunoblotting [11]. Immunoblotting and immunohistochemistry was performed as previously described [11], and primary antibodies are listed in Supplementary Table 1.

### *In vivo* protocol and tumourigenicity and metastasis assays

IGR-CaP1 cells (2×10^6^ tumor cells/ mouse in 50% v/v Matrigel^®^, BD Biosciences) were subcutaneously injected into 6-8 week-old male nude mice in the right flank and measured as previously described [39]. When tumor reached an approximate volume of 1 cm^3^ (72±7 days after tumour cells injection), surgical extirpation of subcutaneous tumours was performed after previous administration of i.p. anesthesia (Ketamine 100 mg/kg and Xylacine 10 mg/kg). After surgery, analgesic (Meloxicam 5 mg/kg) and antibiotic were administrated i.p. (Enrofloxacin 85 mg/kg). During a week, antibiotic (Enrofloxacin 85 mg/kg) and analgesic (Tramadol 1 mg/kg) were administrated on the drinking water. Animals were maintained until worsening of healthy parameters was observed. Tumours (after surgical extirpation) and draining lymph nodes (after animal sacrifice (170±6 days after tumour injection)) were both fixed with 4% formaldehyde in PBS and then paraffin sections (5 μm thick) were processed and stained with modified Masson trichrome (MT).

### Cell growth measurement

Cell growth kinetics were determined by counting the number of viable cells at regular intervals. After seeding in triplicate at 10,000 cells/ well in 96-well plates in standard culture medium, cells were trypsinized daily, stained with trypan blue and counted with Neubauer chambers. The doubling time was calculated using the regression equation of the curve.

### Cell aggregation assay

Single-cell suspensions were obtained and aliquots of 1 ml containing 5×10^5^ cells were allowed to associate in medium containing 5% FBS with gentle rotation (80 rpm) at 37°C for 1 h. After the incubation, three 25-μl aliquots from each sample were spotted onto a microscope slide and dried 1 hour at room temperature. The slides were fixed overnight in a closed container with 37% formaldehyde in a bottom reservoir. Immediately before microscopic evaluation, 25 μl of PBS were placed onto each spot to facilitate the viewing. The total number of cells and the number of cells in aggregates were counted. The percentage of cells in aggregates (number of cells in aggregates/ total number of cells x 100) and the average number of aggregates cells were calculated for individual samples. Each assay was performed in triplicates. Results are presented as means ± SD of the proportion of the % of aggregated PCa cells.

### Clonogenic survival and growth assay

Confluent cells were harvested to obtain a single-cell suspension and plated at a low density in triplicates (3,000 viable cells per well) in a 6-well culture plate. Two weeks later, the cells were fixed with 4% PFA in PBS at 4°C for 5 min and stained with crystal violet, and colonies were counted. Results are presented as the mean percentage of colony forming units obtained for each condition; error bars indicate SD.

### Tumor-endothelial cell adhesion assay

12-well plates were seeded with 4×10^4^ BAEC and allowed to grow and form a complete monolayer. PCa cells were suspended in DMEM medium containing 20% of FBS and added (1.5×10^5^ cells / well) to the BAEC monolayer for 12 h. After the incubation, cells were washed twice with warm free-FBS medium and fixed with 4% PFA in PBS at 4°C for 5 min. Cells were washed with PBS and 14 random fields were photographed under bright and fluorescence microscopy to determine the number of GFP positive cells / field adhered to the BAEC monolayer.

### Scratch-wound assay

Migration was evaluated by means of the scratch-wound assay. Thus, IGR-CaP1 cells (1.5×10^4^) were plated on 96 wells dishes and were allowed to grow to confluence in RPMI containing 10% FBS for 48h. A scratch was performed using a sterile 10-μl pipette tip. The wounded monolayers were washed twice with PBS to remove non-adherent cells and incubated in RPMI without FBS. ImageJ software was used to determine the percentage of reduction of the wound width at 48 h vs. 0 h [width _0 h_ – width _48 h_ / width _0 h_ x 100] (wound closure).

### Analysis of F-actin reorganization as filopodia formation by immunofluorescent staining

Cells were plated on poly-L-lysine-coated glass coverslips for 48 h and fixed with 4% PFA – 4% sucrose. Then cells were permeabilized with 0,05% Triton X-100, incubated with glycine 100 mM in PBS, blocked with BSA 3% in PBS overnight and subsequently stained with rhodamine-conjugated phalloidin. After rinsing with PBS three times, slides were mounted with Mowiol and were examined and photographed under confocal microscope (Olympus). Number of filopodia per cell was counted in at least 25 cells per condition; the results are shown as the mean of three independent experiments.

### Resistance to anchorage-dependent cell death (Anoikis)

To prepare polyHEMA-coated dishes, 0.8 ml of polyHEMA solution (10 mg/ml in 95% ethanol) were placed onto 60-mm Petri dishes and dried in a tissue culture hood. The polyHEMA coating was repeated twice, followed by three washes with PBS. Anoikis was induced by culturing 1×10^6^ cells in polyHEMA-coated 60-mm dishes in RPMI 1640 supplemented as described above.

### Analysis of cell cycle

The percentage of cells in each cell cycle phase was determined by flow cytometry (FACSAria II cell sorter - BD Bioscience). Cells grown for 12, 24 or 48 h in suspension (polyHEMA-coated dishes) were trypsinized, suspended in culture medium, centrifuged at 150xg for 5 min, and fixed with cold 70% ethanol for at least 2 hours on ice. The fixed cells were centrifuged, washed with PBS and stained with propidium iodide staining solution (0.1% Triton X-100, 0.02% m/v DNase-free RNase A and 0.002% m/v propidium iodide in PBS) 15 min at 37 °C. The stained cells were analyzed by flow cytometry. Data was analysed using FlowJo 8.7 software.

### Statistic analysis

Data are presented as mean ± standard deviation (S.D.) of at least three separate experiments in triplicate. Comparisons between two groups were performed by using paired Student's t-test. Differences were considered significant when p values were less than 0.05 as shown in all figures when needed.

D.J.L., M.K, and D.C. are researchers of Consejo Nacional de Investigaciones Científicas y Técnicas (CONICET-Argentina). L.D.G., I.G-P, F.M.J. are post-graduate fellows from ANPCyT or/and CONICET. A.C. is a researcher of the Institut National de la Santé et de la Recherche Médical (INSERM-France).

## SUPPLEMENTARY MATERIALS FIGURES AND TABLES


