# Supplementary material for: Stable and high expression of Galectin-8 tightly controls metastatic progression of prostate cancer
Source: Oncotarget. 2017 May 18;8(27):44654–68. doi: 10.18632/oncotarget.17963 (PMC5546508; doi:10.18632/oncotarget.17963)
Supplement: Supplementary file 1 [file oncotarget-08-44654-s001.pdf]

Stable and high expression of Galectin-8 tightly controls metastatic progression of prostate cancer

SUPPLEMENTARY MATERIALS

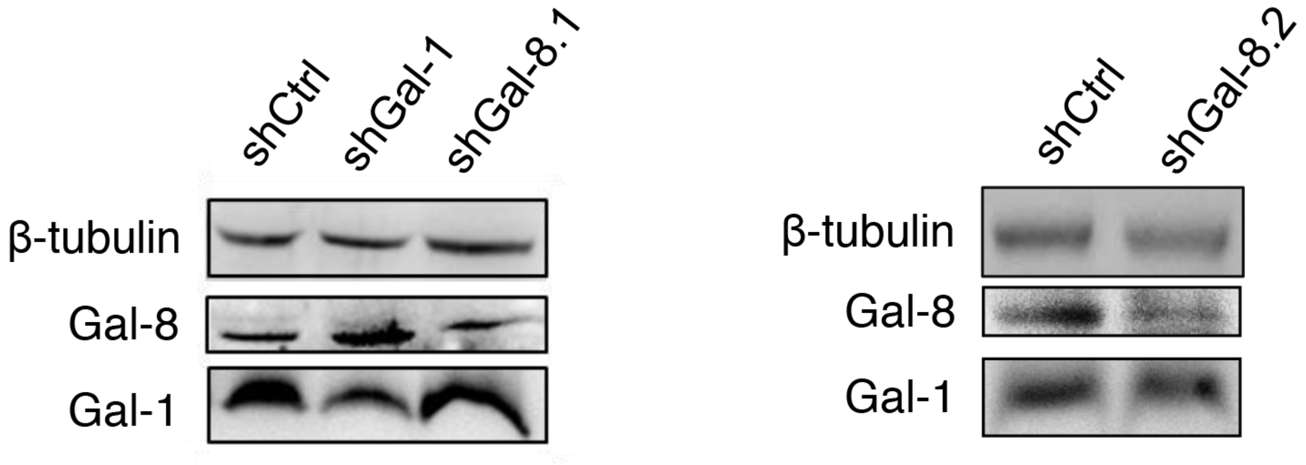

Supplementary Figure 1: Silencing of Gal-8 in IGR-CaP1. Western blotting to analyze the Gal-8 or Gal-1 expression in IGR-CaP cells transduced with a control-shRNA (shCtrl) or two different Gal-8-shRNA -expressing LV (shGal-8.1 or shGal-8.2), and Gal-1-shRNA-expressing LV (shGal-1).

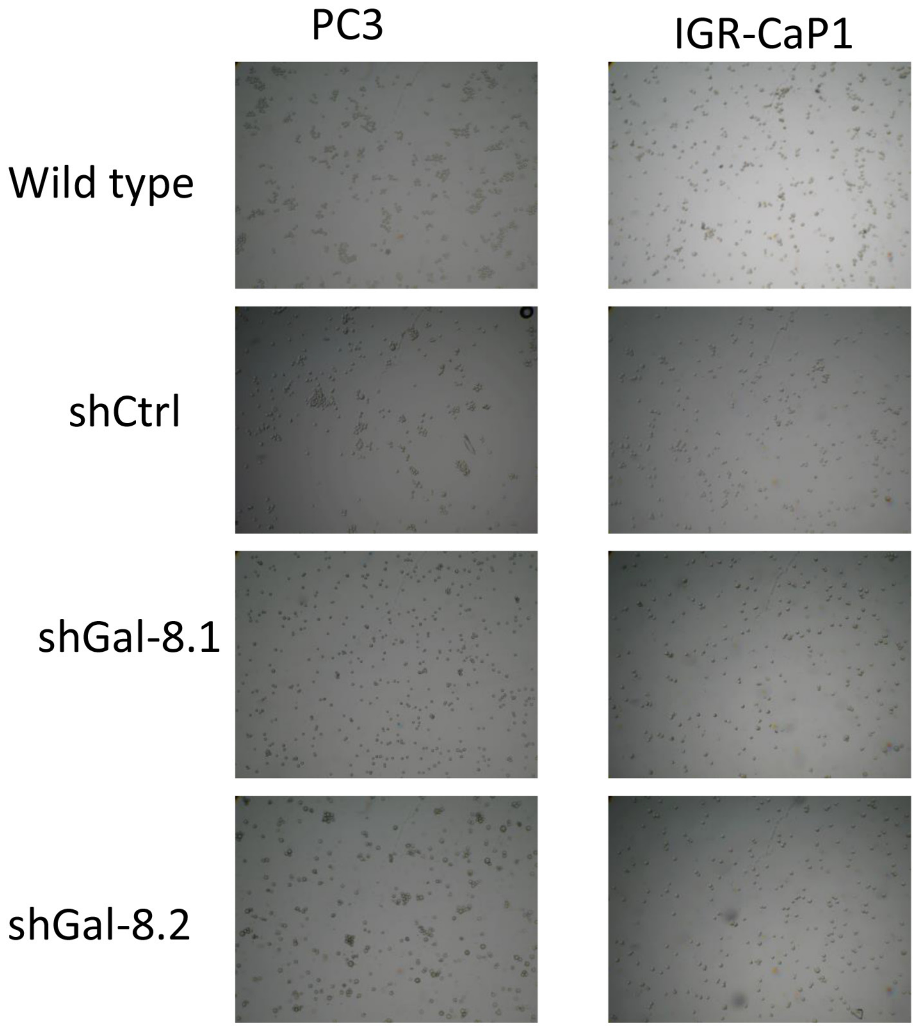

Supplementary Figure 2: Silencing of Gal-8 decreases homotypic aggregations of IGR-CaP1 and PC3.

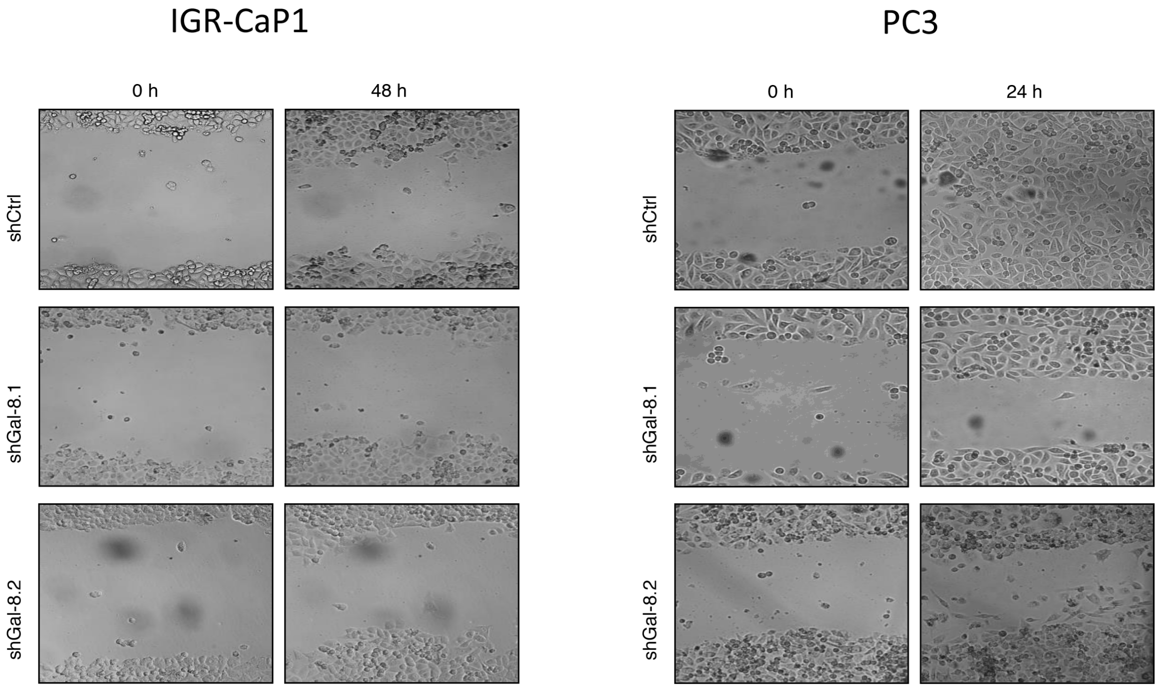

Supplementary Figure 3: Silencing of Gal-8 decreases migration capacities of IGR-CaP1 and PC3.

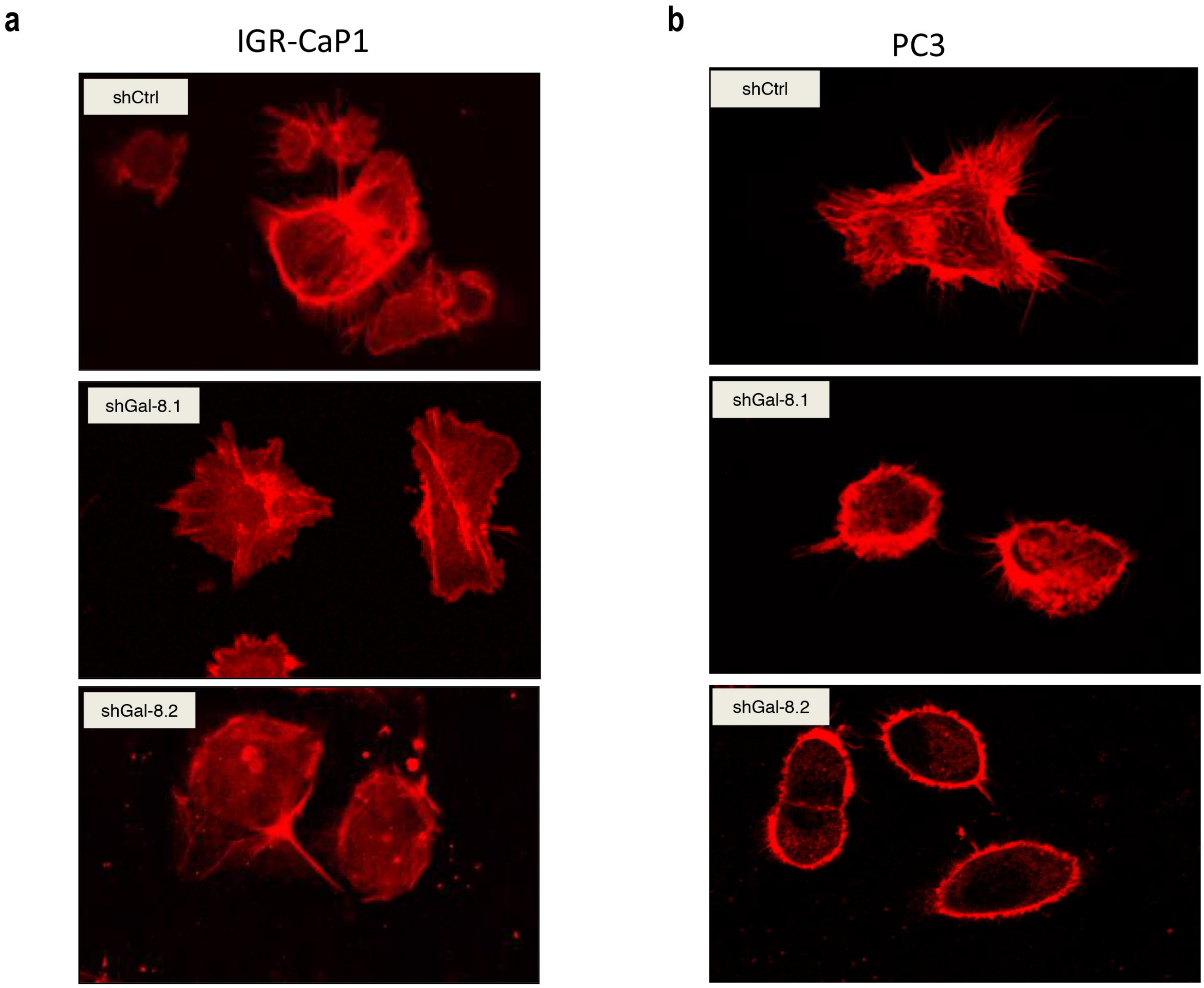

Supplementary Figure 4: Gal-8 knock-down decreases the cytoskeleton reorganization. Representative photographs of the analysis of filopodia formation by staining the Actin cytoskeleton with Rhodamin-conjugated phalloidin on PCa cells lines. **(a)** IGR-CaP1 and **(b)** in PC3.

Supplementary Table 1: Antibodies used to determine galectins, apoptosis markers and E-Cadherin expressions

Protein	Antibody catalog #	Dilution
β-actin	H196, Santa Cruz	1:1000
β-tubulin	H-235, Santa Cruz	1:200
Galectin-1	H-45	1:500
Galectin-3	H-160, Santa Cruz	1:400
Galectin-8	AF1305, R&D	1:600
Caspase-3	9661, Cell signaling	1: 1000
Cleaved Caspase-3	9662, Cell signaling	1:1000
PARP	H-250, Santa Cruz	1:100
E-cadherin	610182, BD	1:2500
